# A Fluorescence-Based Thermal Shift Assay Identifies Inhibitors of Mitogen Activated Protein Kinase Kinase 4

**DOI:** 10.1371/journal.pone.0081504

**Published:** 2013-12-05

**Authors:** Sankar N. Krishna, Chi-Hao Luan, Rama K. Mishra, Li Xu, Karl A. Scheidt, Wayne F. Anderson, Raymond C. Bergan

**Affiliations:** 1 Department of Medicine, Northwestern University, Chicago, Illinois, United States of America; 2 Department of Molecular Biosciences, Northwestern University, Chicago, Illinois, United States of America; 3 Department of Chemistry, Northwestern University, Chicago, Illinois, United States of America; 4 Department of Molecular Pharmacology and Biological Chemistry, Northwestern University, Chicago, Illinois, United States of America; 5 High Throughput Analysis Laboratory, Northwestern University, Chicago, Illinois, United States of America; 6 Center for Molecular Innovation and Drug Discovery, Northwestern University, Chicago, Illinois, United States of America; 7 The Robert H. Lurie Cancer Center, Northwestern University, Chicago, Illinois, United States of America; Albert-Ludwigs-University, Germany

## Abstract

Prostate cancer (PCa) is the second highest cause of cancer death in United States males. If the metastatic movement of PCa cells could be inhibited, then mortality from PCa could be greatly reduced. Mitogen-activated protein kinase kinase 4 (MAP2K4) has previously been shown to activate pro-invasion signaling pathways in human PCa. Recognizing that MAP2K4 represents a novel and validated therapeutic target, we sought to develop and characterize an efficient process for the identification of small molecules that target MAP2K4. Using a fluorescence-based thermal shift assay (FTS) assay, we first evaluated an 80 compound library of known kinase inhibitors, thereby identifying 8 hits that thermally stabilized MAP2K4 in a concentration dependent manner. We then developed an *in vitro* MAP2K4 kinase assay employing the biologically relevant downstream substrates, JNK1 and p38 MAPK, to evaluate kinase inhibitory function. In this manner, we validated the performance of our initial FTS screen. We next applied this approach to a 2000 compound chemically diverse library, identified 7 hits, and confirmed them in the *in vitro* kinase assay. Finally, by coupling our structure-activity relationship data to MAP2K4's crystal structure, we constructed a model for ligand binding. It predicts binding of our identified inhibitory compounds to the ATP binding pocket. Herein we report the creation of a robust inhibitor-screening platform with the ability to inform the discovery and design of new and potent MAP2K4 inhibitors.

## Introduction

Prostate cancer (PCa) is the most common cancer type among men in the United States. Its spread from the primary prostate organ to other parts of the body through the process of metastasis constitutes the second highest cause of death due to cancer among males in the United States[1]. The metastatic progression of prostate cancer (PCa) cells leads to cell detachment and invasion, and eventually to movement of cells beyond the prostate[2]. If it were possible to inhibit the metastatic spread of PCa cells by therapeutically targeting proteins driving that process, then this disruption should result in a substantial decrease in cancer mortality.

**Figure 1:**
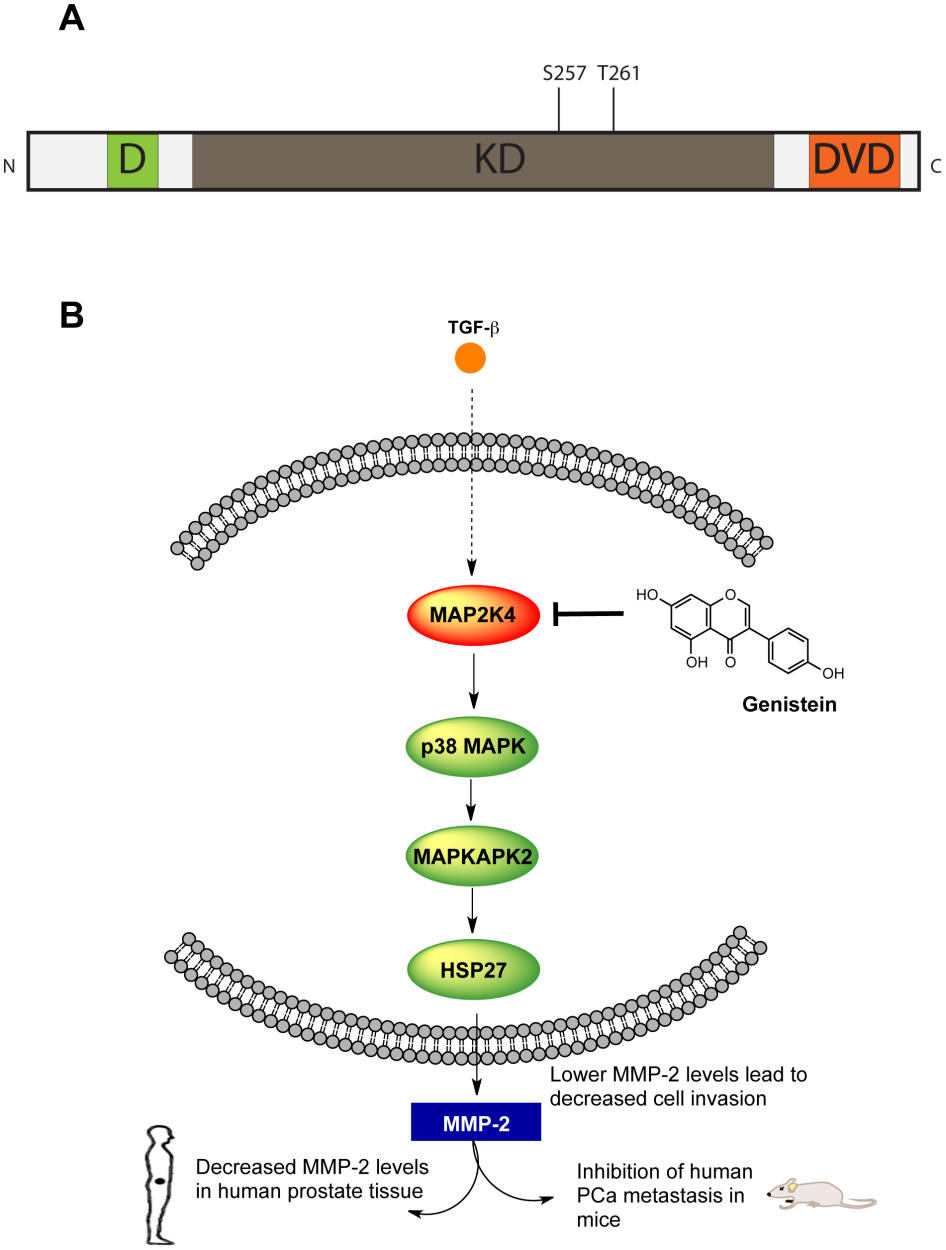
MAP2K4's role in prostate cancer metastasis. **A**. The domains of MAP2K4. MAP2K4 has three distinct domains; the kinase domain (KD) is involved in the actual kinase activity, the docking domain (D) mediates binding to downstream MAPKs and the domain of versatile docking (DVD) mediates interactions with upstream activators. **B**. Genistein inhibits MAP2K4 in human prostate cancer cells, thereby inhibiting phosphorylation of downstream effector proteins leading to down-regulation of MMP-2 expression *in vitro* and in prostate tissue in humans, inhibition of cell invasion, and inhibition of human prostate cancer metastasis in mice.

We have previously identified mitogen-activated protein kinase kinase 4 (MAP2K4; also known as MEK4, MKK4 or SEK1), a 399 amino acid protein, as a driver of metastatic transformation in human PCa, and as an important target of small molecule therapeutics designed to inhibit metastasis [3]. MAP2K4 is a dual-specificity kinase, i.e., it phosphorylates serine/threonine as well as tyrosine residues, and it constitutes a second tier signaling protein of the canonical three-tier MAP kinase cascade [4]. While the central kinase domain (KD), residues 102-367, is responsible for its catalytic activity, MAP2K4 also contains distinct C- and N- terminal domains. The C-terminal domain of versatile docking (DVD), residues 364-387, binds upstream MAP kinase kinase kinases (MAP3K1/MAP3K11) which in turn phosphorylate MAP2K4 (**Figure 1A**) [5] at serine 257 and threonine 261, thereby regulating MAP2K4 kinase activity. The N-terminal D domain, residues 37–52, contains a conserved docking site that is required for substrate recognition. MAP2K4 in turn phosphorylates and activates two classes of downstream MAP kinases: c-Jun N-terminal kinases (JNK1-3) and p38 mitogen activated kinases (p38α-γ MAPK) [6], [7]. Crystal structures of MAP2K4 (PDB: 3ALN, 3ALO) show that it conforms to the typical bilobal kinase fold of a N-terminal beta sheet rich region, a mostly alpha helical C-terminal portion and a cleft in between forming the ATP binding site [8].

In humans, increased expression of MAP2K4 is found in invasive cancer lesions in the prostate tissue of men with PCa, as is MMP-2, and their presence portends the development of metastasis[9]-[11]. MMP-2 is a protease that acts to degrade the extracellular matrix, and thus it greatly facilitates the ability of cancer cells to invade out of the prostate gland and to spread throughout the body[12]. Through an extensive series of *in vitro* studies, employing differential engineered expression of MAP2K4 and associated use of small molecule inhibitors, we have demonstrated that MAP2K4 increases the expression of MMP-2 and cell invasion in human PCa cells, and that it does so by activating the p38 MAPK pathway (**Figure 1B**) [3], [13]–[15]. Importantly, we have shown that MAP2K4 is targeted by the small molecule genistein (4,5,7-trihydroxyisoflavone) and that genistein inhibits the metastasis of human PCa cells orthotopically implanted into mice [16]. Finally, we showed that prospective administration of genistein to humans selectively decreases MMP-2 expression in prostate tissue [3]. Importantly, MAP2K4 appears to have a similar pro-invasion/pro-metastatic role in several other cancer types, including breast and pancreatic cancer [17]. Together, these studies identify MAP2K4 as an important regulator of human PCa metastasis, and demonstrate that small molecules can target it with therapeutic efficacy in both pre-clinical models as well as in early phase human trials. Furthermore, this key pathway appears to play a similarly important role in other cancer types [17].

The ability of MAP2K4 to be therapeutically modulated by the natural product genistein makes it a promising candidate for anti-metastatic intervention. However, genistein is a less than ideal small molecule lead compound. It is a natural product and it exerts a wide range of biological effects. In particular, genistein is considered a broad-spectrum tyrosine kinase inhibitor, has poor potency, and has serious undesired side effects, including estrogenic receptor stimulation [18]–[20]. Recognizing the therapeutic potential of MAP2K4 in PCa, we sought to identify small molecule inhibitors that target it. Herein, we have developed a MAP2K4 fluorescence-based thermal shift (FTS) assay, and have used it to identify MAP2K4 binding compounds. We have also developed a MAP2K4 *in vitro* kinase assay in order to validate FTS findings. We did so across two separate chemical libraries, each with distinct characteristics. In this manner, we validated a biochemical MAP2K4 inhibitor-screening platform. An *in silico* model of MAP2K4 docked with the identified inhibitors was constructed and serves as a tool to guide future drug discovery activities. These findings provide a highly informative platform for the design and discovery of small molecule inhibitors of MAP2K4, a critical kinase in the metastatic pathway of human PCa, likely so for other cancers, and potentially important in other disease processes.

## Materials and Methods

### Cloning, expression and purification of MAP2K4

Human MAP2K4-EE (residues 37–399; **Figure S1**), containing mutations that mimic a constitutively active/phosphorylated state, S257E and T261E, was cloned into the pMCSG7 vector as previously described [21] and transformed into Single Step KRX Competent *E. coli* cells (Promega Inc) containing the pGro7 plasmid (Takara, Japan). The KRX cells contained a T7 Promoter that regulated expression of the inserted protein. L-arabinose (0.2%) was added to Terrific Broth growth media upon inoculation to express the molecular chaperones GroEL and GroES from the pGro7 plasmid. Cells were grown at 37°C until an OD_600_ of 0.6–0.8 was reached. At which point, the temperature was reduced to 25°C and protein over-expression induced by the addition of 0.1 mM isopropyl-1-thio-D-galactopyranoside (IPTG) and 0.25% w/v L-Rhamnose. After overnight growth, cells were harvested by centrifugation, resuspended in a buffer containing 10 mM Tris-HCl pH 8.3, 500 mM NaCl, 10% glycerol, and 5 mM β-mercaptoethanol and lysed by sonication. The protein was purified by Ni-NTA affinity chromatography (500 mM Imidazole step elution) and further purified using a size-exclusion column, into a buffer containing 10 mM Tris-HCl pH 8.3, 500 mM NaCl, 5 mM β-mercaptoethanol and 1 mM TCEP-HCl. MAP2K4-EE was concentrated using a Centrifugal Filter Device (Millipore) with a 10,000 Da molecular weight cutoff typically to 5 mg/ml. Protein concentration (A_280_) was measured using a Nanodrop 1000 (Thermo Scientific) and the protein was aliqouted, frozen in liquid nitrogen and stored in a −80°C freezer. Human MAP2K4-KD (80–399; **Figure S1**) was expressed and purified in identical fashion but with the gene of interest being cloned into the pMCSG28 vector with a C-terminal 6X histidine tag instead.

### Fluorescence-based thermal shift (FTS) assay

Small molecule screening by FTS assay was performed as described previously[22], [23] but with modifications, using an Echo550 (Labcyte) for compound transfer and a Mosquito (TTP Labtech) to dispense protein into 384 well PCR plates. Purified MAP2K4-EE was appropriately diluted in a buffer containing 100 mM Hepes, pH 7.5, 150 mM NaCl. All assay experiments used 2 µg protein per well and 5 nl 5000X Sypro Orange (Invitrogen) upto a total volume of 10 µl, with a resultant protein concentration of 4.65 µM. The ScreenWell Kinase Inhibitor library (Enzo Life Sciences Cat# BML-3328) and the Spectrum Collection (Microsource Discovery Systems Inc) were supplied at 10 mM concentration in DMSO. The PCR plates were sealed with optical seal, shaken, and centrifuged after protein and compounds were added. Thermal scanning (10 to 95°C at 1.5°C/min) was performed using a real-time PCR setup (CFX384 - Biorad Laboratories) and fluorescence intensity was measured after every 10 seconds. Curve fitting, melting temperature calculation and report generation on the raw FTS data were performed using software developed by us. Initial screening with the 2000 compound Spectrum Collection was carried out in a pooled fashion. Specifically, 3 compounds were added at 10 µM concentration to each well and wells with positive Tm shift >2°C were then dissected to identify the responsible compound.

### In-vitro kinase assay

*In vitro* kinase assays utilized a N-terminal GST tagged MAP3K1 activated murine MAP2K4 (Millipore; Catalog # 14–377), MAP2K4-AC (**Figure S1**). In addition to activated MAP2K4, the *in vitro* kinase assay used either kinase dead JNK1-K55M (Abcam # ab95248) or kinase dead p38α MAPK-K53A (generously provided by Dr. Martin Watterson, Northwestern) as the substrate, ATP (Cell Signaling) and kinase assay buffer (25 mM Tris-HCl (pH 7.5), 5 mM beta-glycerophosphate, 2 mM dithiothreitol (DTT), 0.1 mM Na3VO4, 10 mM MgCl2). Quercetin and daidzein were purchased from Enzo Life Sciences. Geraldol, 2′,4′-dihydroxychalcone and 3,2′-dihyrdoxy-4,4′,6′-trimethoxychalcone were purchased from Indofine Chemical Company. All other compounds were purchased from Sigma-Aldrich. To tubes containing 13 µl of 1.5X buffer on ice the assay reagents were added as indicated for a final reaction volume of 20 µL: 25 ng of MAP2K4-AC (stocks of 25 ng/uL were prepared and aliquoted from a 10 ug vial after the supplier concentration was verified using a Nanodrop 1000), inhibitors as denoted (from DMSO stock solutions), 1 µM final ATP, and 1 µg of JNK1 or p38 MAPK. All reactions were incubated simultaneously for 5 minutes at 30°C, after which 20 µl of Laemlli buffer (Biorad) containing 5% v/v 2-mercaptoethanol was added and tubes heated to 95°C for 5 minutes and then placed on ice. All kinase assays were performed twice at separate times.

### Western Blotting and densitometry analysis

Twenty µl of reaction mixtures were separated by sodium dodecyl sulfate-polyacrylamide gel electrophoresis using precast 4–20% gradient gels (Biorad), per the manufacturers' instructions, and transferred onto 0.45 µm nitrocellulose membranes (Biorad). After blocking with 5% bovine serum albumin (BSA) the membranes were incubated overnight (14–18 hours) at 4°C with anti-phospho JNK1+JNK2 (Abcam # ab4821) or anti-phosphorylated p38 MAPK (Cell Signaling # 4631S) (1∶500 dilution in 5% BSA). Bands were visualized with the aid of chemiluminescence reagents (GE Healthcare) after hybridization with a HRP conjugated rabbit secondary antibody (GE Healthcare). The blots were then stripped at 55°C for 20 minutes in a buffer (100 mM 2-mercaptoethanol, 2% sodium dodecyl sulfate, and 62.5 mM Tris–HCl at pH 6.7) before being re-probed and visualized by antibodies to total JNK (Abcam #ab85139) or p38 MAPK (Cell Signaling #9212S), to thereby establish equal protein loading. The bands were quantified using ImageJ software; normalized inhibitor-response data were plotted using Prism 6 to determine relative IC50 values.

### In silico docking and binding energy calculations

*In silico* docking analyses with a MAP2K4 crystal structure (PDB: 3ALO), amino acids 80–399 (**Figure S1**) were performed using Schrodinger Suite 2011, using the Maestro interface. Docked poses were generated using the Induced Fit protocol, which aims to improve the docking of the ligands, in which the receptor adjusts in the presence of the ligand [24], [25]. A constrained minimization of the receptor followed by Glide docking of the ligands using a softened potential was performed first. Then a select set of docked poses was passed on to the Prime module for refinement. After side-chain reorientation and minimization using Prime, the best receptor structures for each ligand are passed back to Glide for extra precision docking. Binding energies were computed using the Molecular Mechanics Generalized Born model and Solvent Accessibility (MM-GBSA) method, and the highest scoring poses for each ligand were selected. Figures were generated using PyMol.

## Results and Discussion

### The fluorescence-based thermal shift assay is tractable for use with MAP2K4

The FTS assay operates on the principle that ligand binding alters thermal stability of proteins [26], [27]. Note that the FTS assay has been referred to by several different names, including differential scanning fluorimetry (DSF) and ThermoFlour™ assays. We have elected to use FTS because it constitutes an accurate description of the actual assay and has been used previously by others. ThermoFlour™ is a trademark, while DSF incorrectly draws a parallel with the technique of differential scanning calorimetry (DSC). FTS provides a measurement of a temperature shift, while DSC provides a measure of required heat for a given temperature shift. The FTS setup allows one to monitor protein denaturation upon heating via fluorescent-based detection. A fluorescent dye based probe is used that preferentially binds the hydrophobic regions of a protein, which are increasingly exposed during protein denaturation. When coupled to a real-time PCR setup, monitoring the change in fluorescence provides a thermal melting curve or thermogram. The mid-point of the melting curve, i.e., the temperature at which 50% of the protein has denatured, is designated as the melting temperature, Tm, and is a measure of the protein's inherent thermal stability [22]. A ligand bound to a protein, e.g. to its active site, has the propensity to increase its thermal stability (and hence it’s Tm) through newly formed ligand-protein interactions. In the case of kinases, this difference in melting temperature (ΔTm) of the protein and of the ligand-protein complex has been shown previously to correlate to measures of the ligand's concentration and binding affinity [23]. In this manner, a melting curve is generated, the Tm determined, and changes in Tm (ΔTm) induced by prospective binding ligands can be calculated.

**Figure 2:**
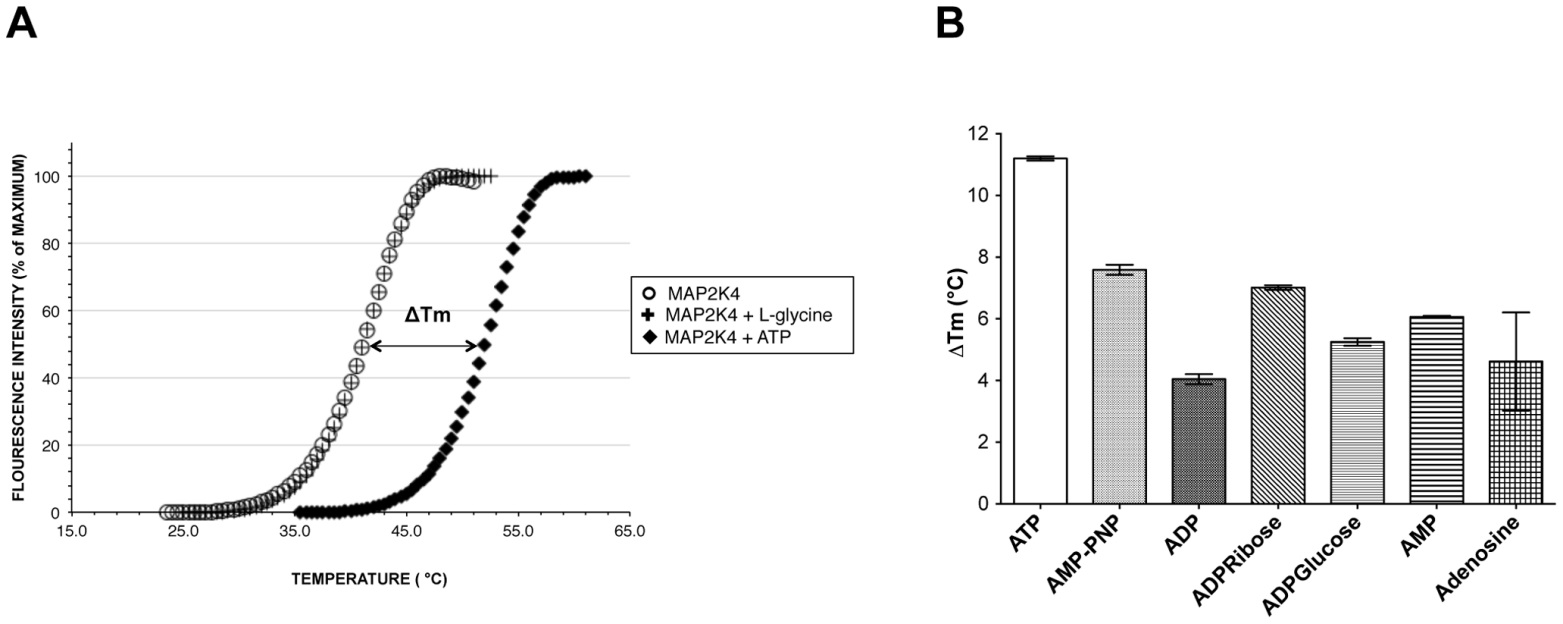
The fluorescence-based thermal shift assay is tractable for use with MAP2K4. **A**. MAP2K4 undergoes a two-phase transition denaturation profile. MAP2K4 was subjected to FTS analysis, as described in Methods, in the presence of 1 mM ATP, 1 mM L-glycine, or buffer only. Depicted is a representative denaturation profile. **B**. The effect of adenine moiety containing ligands upon the denaturation profile of MAP2K4. Experiment was performed as in A in the presence of the indicated ligands, all at 1 mM, and the resultant ΔTm values, compared to control (HEPES), determined. Values shown are the mean ± S.D (n = 3).

Not all proteins are amenable to FTS measurements. Non-globular and large multi-domain proteins tend not to exhibit distinct phase transitions during thermal denaturation. To evaluate this in MAP2K4, we first purified recombinant MAP2K4 (MAP2K4-EE **Figure S1**). Then we subjected the kinase to FTS with and without ATP, a known ligand for this protein family. We demonstrated that MAP2K4 went through a distinct two state transition during thermal unfolding when no ligands were present. The mean Tm for MAP2K4-EE was observed to be 40.03°C. One mM ATP induced >10°C shift in Tm (**Figure 2A**), whereas, a non-binder like L-glycine did not induce thermal stability even at high concentration. To determine if FTS could discriminate between different MAP2K4 binding ligands, we examined several other adenine nucleotides, such as adenosine diphosphate (ADP), AMP-PNP, AMP among others (**Figure 2B** and **Figure S2**). Of these, the natural substrate, ATP and the closely related non-hydrolyzable analog AMP-PNP, displayed the highest ΔTm values. Together, these findings demonstrate that MAP2K4 ATP binding site targeted ligands induce increases in Tm, and that even within a given chemical class of compounds (i.e., those containing an adenosine moiety) differences in ΔTm were readily detected. These findings established the assay's tractability, as well as informed the conditions to perform thermal unfolding experiments using potential small molecule inhibitors.

### Identification of small molecule kinase inhibitors that thermally stabilize MAP2K4

The ScreenWell Kinase Inhibitor library from Enzo Life Sciences consists of 80 well-characterized kinase inhibitors. It was utilized as our first screen of choice, and served as a tool to understand the performance of the FTS assay as a primary screen. All 80 compounds were screened by FTS assay at concentrations of 10 µM. Compounds were selected for further analysis if they exhibited a ΔTm at 10 µM ≥2°C and were confirmed if they elicited concentration-dependent increase in ΔTm at 1, 10 and 25 µM.

**Figure 3:**
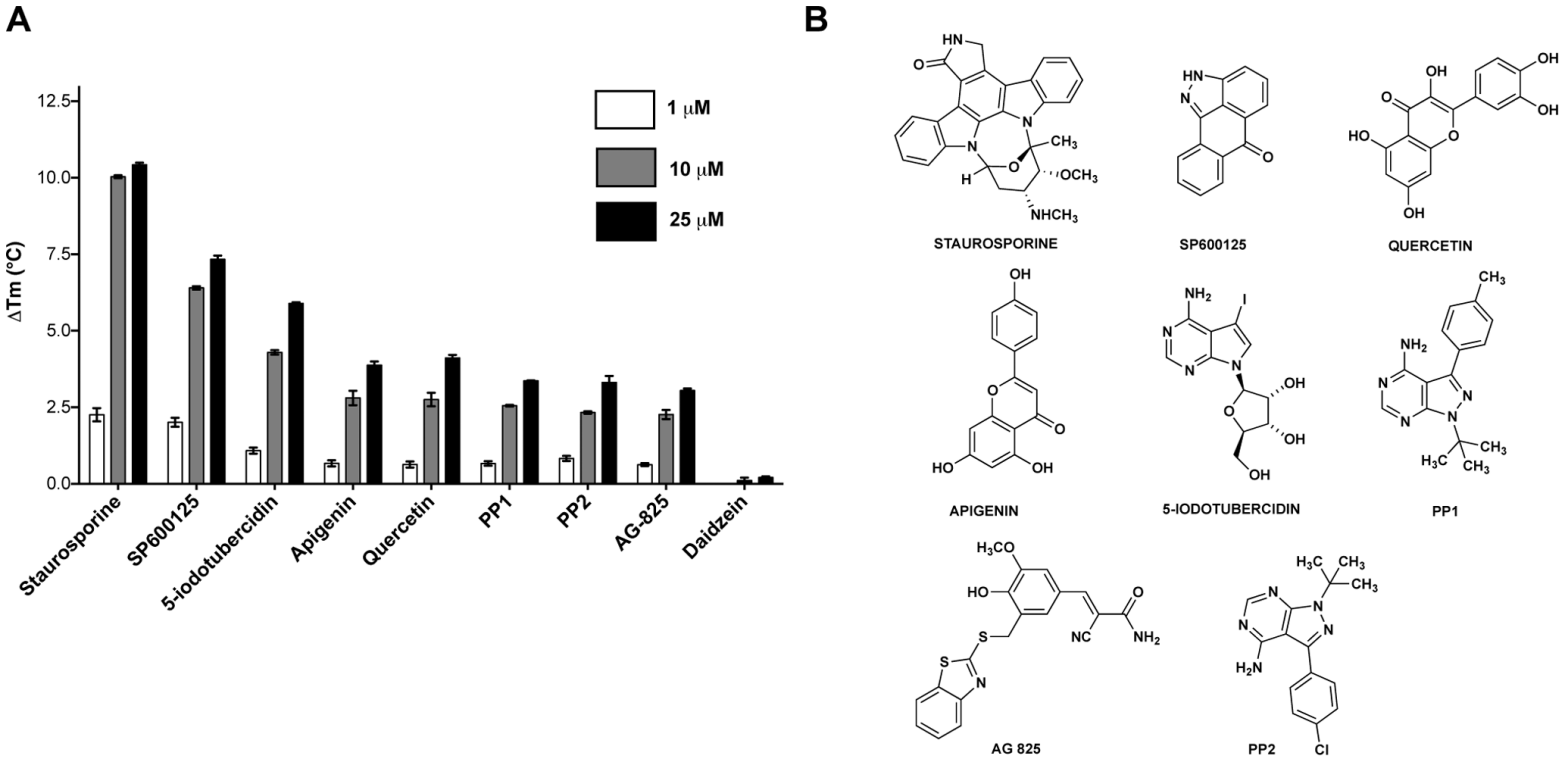
The FTS assay identifies MAP2K4 binders from a library of kinase inhibitors. **A**. Graph of ΔTm values of ScreenWell Kinase Inhibitor library hits determined at compound concentrations of 1, 10 and 25 µM. Daidzein (negative control) is also shown. Values shown are the mean ± S.D (n = 3) **B**. Chemical structures of hits are depicted.

Eight compounds (10% of the library) met our selection criteria, exhibiting ΔTm values ranging from 2.26 – 10.03°C at 10 µM and are listed in **Table 1**. Their chemical structures are depicted in **Figure 3B** and representative raw melting curves are shown in **Figure S3**. Next, we examined FTS denaturation profiles at different compound concentrations, for each of the 8 compounds and ensured they elicited dose-response (**Figure 3A**). The broad-spectrum kinase inhibitor, staurosporine, was found to induce the greatest magnitude of thermal stabilization, with a ΔTm of 10.03°C at 10 µM. Prior findings have shown that staurosporine, an ATP mimetic and a broad-spectrum kinase inhibitor, inhibits MAP kinases with low- to sub-micromolar affinity [28]. Interestingly, the flavonoids apigenin and quercetin showed a concentration dependent thermal shift, with ΔTm values of 2.8°C and 2.75°C, respectively, while the structurally similar flavonoid daidzein had no such thermal stabilization effect (**Table S1**). Both apigenin and quercetin have been shown to inhibit a variety of kinases and to induce a relatively wide array of biological effects [29], [30]. Although structurally similar, daidzein typically lacks such effects and is commonly employed as a negative control in related experiments [31].

**Table 1:** FTS and kinase assay data of hits from the ScreenWell kinase inhibitor library.

Compound	ΔTm at 10 µM (°C)	IC_50_-JNK1 ( µM)	IC_50_-p38 ( µM)
Staurosporine	10.03±0.05	0.6 (0.4 to 1)	0.2 (0.1 to 0.4)
SP600125	6.4±0.06	0.7 (0.3 to 1)	0.4 (0.3 to 0.6)
5-iodotubercidin	4.29±0.07	2.8 (1.2 to 6.3)	0.2 (0.2 to 0.3)
Apigenin	2.8±0.23	20.1 (12.6 to 32.2)	15.9 (10.8 to 23.5)
Quercetin	2.75±0.22	1.7 (1 to 2.8)	2.0 (1.4 to 3.0)
PP1	2.55±0.03	16.9 (9.9 to 28.8)	13.8 (5.7 to 33.3)
PP2	2.33±0.04	22.3 (11 to 45.2)	39.9 (13.2 to 121)
AG825	2.26±0.15	25.0 (11.5 to 54.6)	28.5 (15.8 to 51.6)

Data shown as Mean ± SD with n = 3 for ΔTm. IC_50_ values are Mean (95% confidence intervals) calculated by fitting data averaged from replicates performed twice at separate times.

Also of interest, inhibitors of kinases related to MAP2K4 were identified in our FTS screen. These include, 5-iodotubercidin, an inhibitor of the MAP kinase, ERK2, and SP600125, an inhibitor of the MAP kinases, JNK1 and JNK2, which presented ΔTm values of 4.3°C and 6.4°C at 10 µM respectively (**Table 1**). Other compounds acting against less similar kinases, such as the HER-2/neu inhibitor, AG 825, and the Src-family inhibitors, PP1 and PP2, also thermally stabilized MAP2K4, by 2.3, 2.5 and 2.3°C at 10 µM respectively, albeit to a lesser degree compared to inhibitors of structurally similar kinases. Similar results were obtained when the non-phosphorylated kinase domain construct, MAP2K4-KD (**Figure S1**) was screened with this library with the 8 selected hits demonstrating comparable dose-dependent response as with MAP2K4-EE (**Figure S4**).

It is to be noted that this was a library of kinase inhibitors, most of which are known or presumed to bind the ATP pocket, a conserved feature among kinases. The promiscuity of kinase inhibitors is recognized, and is generally attributed to similar inhibitor-ATP pocket interactions [23]. Therefore, in the context of this, we used a deliberately high threshold for the MAP2K4 FTS assay to identify only a subset of the 80 compounds as potent MAP2K4 binders. As our primary purpose to screen such a library was to establish the viability of the assay and its downstream validation we did disregard compounds that elicited ΔTm between 1-2°C shift at 10 µM (**Table S1**). Therefore in a HTS scenario while screening a larger and structurally diverse library, even such compounds could be viable starting points for novel chemistry.

### Inhibition of MAP2K4 kinase activity validates FTS assay hits

**Figure 4:**
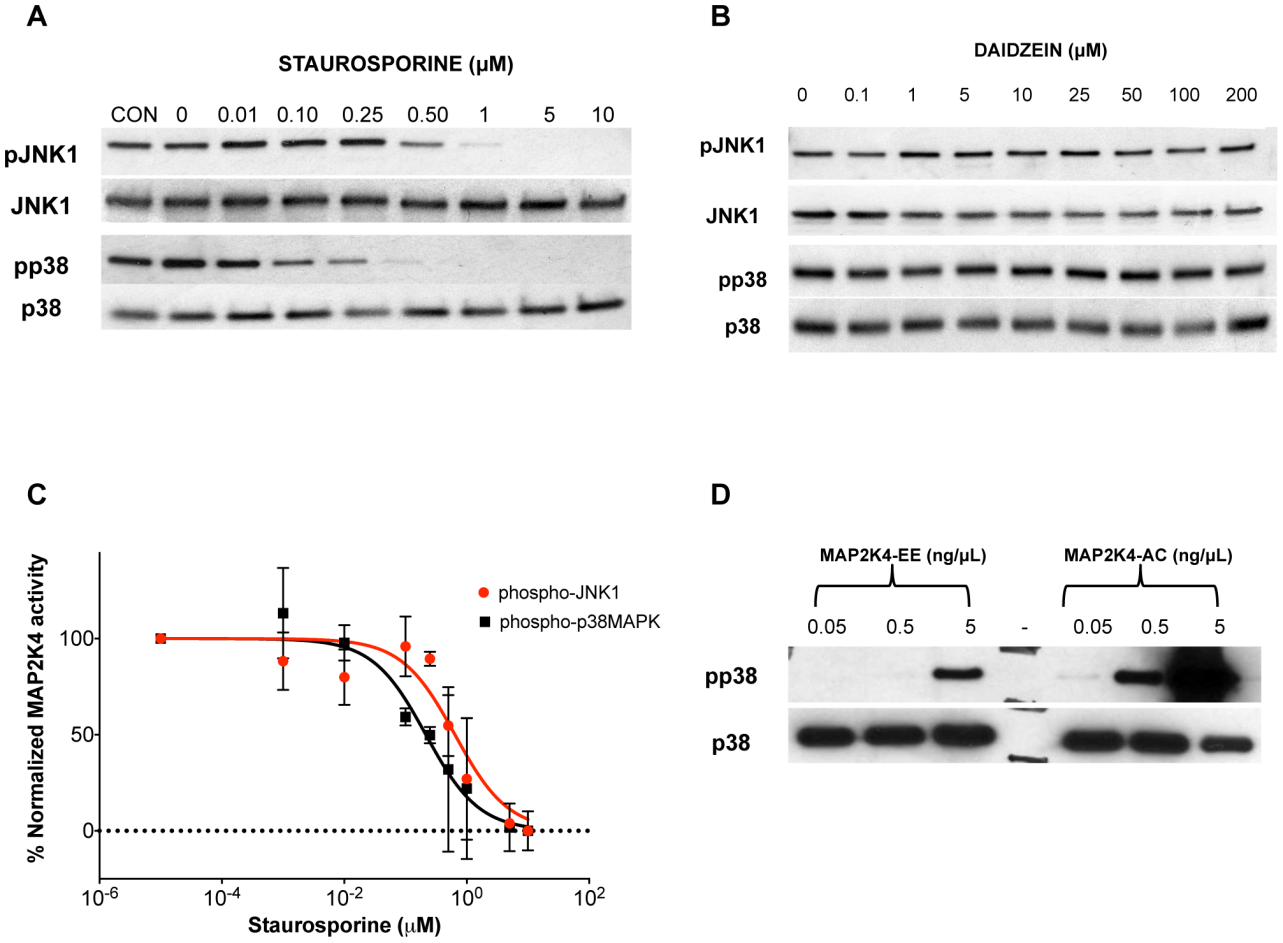
Kinase Assay validates FTS hits as inhibitors. MAP2K4 kinase assays were performed as described in Methods using either p38 MAPK (K53A) or JNK1 (K55M) as substrates, and was done using a range of concentrations of either staurosporine **A** or daidzein **B**. Representative phosphorylated JNK1 and phosphorylated p38 MAPK and total JNK and p38 MAPK Western blots are depicted. **C**. A plot of densitometric measurement of bands from separate experiments utilizing Staurosporine (2X each on p38 and JNK1 substrates) is depicted with intensity measurements shown as mean ± S.D along with the curve fit used for determining the IC_50_ values; Red – JNK1 and Black – p38MAPK. **D**. 3 indicated concentrations of either MAP2K4-EE or MAP2K4-AC were employed in the kinase assay and the western blot performed as before.

In intact cellular systems, MAP2K4 activates c-Jun N-terminal kinases, JNK1-3, and p38 mitogen-activated protein (MAP) kinases, p38α and p38β [32]. MAP2K4-mediated activation results from dual phosphorylation at Thr-180 and Tyr-182 on p38 MAP kinases [33] and at Thr-183 and Tyr-185 on JNKs [34]. Thus, an *in vitro* kinase assay incorporating MAP2K4, ATP and either p38 MAP kinase or JNK would emulate the *in vivo* biologically relevant scenario. We therefore developed an assay; using activated murine MAP2K4 (MAP2K4-AC in **Figure S1**) with kinase-dead forms of either JNK1 (K55M) or p38α MAPK (K53A). In **Figure 4A**, representative results from the kinase assay using staurosporine are depicted. Staurosporine effectively inhibits MAP2K4-mediated phosphorylation of both JNK1 and p38 MAP kinase at sub-micromolar concentrations. Densitometry analysis was performed on all kinase assays to quantify the inhibitor concentration at which the half-maximal normalized response (IC_50_) was calculated. A representative graph of such analysis from kinase assays employing both protein substrates and Staurosporine is depicted in **Figure 4C**. Daidzein does not inhibit MAP2K4 activity at concentrations of up to 200 µM. In **Figure 4B**, representative kinase assay results using daidzein, which does not thermally stabilize MAP2K4, are depicted.

In a similar fashion, each of the 8 compounds identified as hits in the FTS assay were evaluated in the *in vitro* MAP2K4 kinase assay, and all were found to inhibit MAP2K4-mediated phosphorylation of JNK1 and p38 MAP kinase *in vitro* (**Table 1** **, Figure S5, and Figure S6**). It is notable that staurosporine, SP600125 and 5-iodotubercidin, all compounds that stabilized MAP2K4 by >4°C, inhibited kinase activity with half maximal inhibitory concentrations (IC_50_) in the sub-micromolar to low-micromolar range. A comparison between the IC_50_ values derived from *in vitro* kinase assays using p38 and JNK1 showed strong correlation between them (Pearson coefficient, r = 0.905, P-value = 0.002). The strong similarities in inhibitor behavior across both protein substrates permitted us to refine the validation step and focus future screening efforts on the less expensive p38 MAPK system.

Mutation to a negatively charged aspartate or glutamate of the catalytically important serine and threonine residues in MAP2K4 leads to a constitutively active form and is thought to mimic a phosphorylated form of the enzyme [3]. We employed the kinase assay to verify if the recombinant human MAP2K4-EE construct was capable of phosphorylating its substrate in-vitro (**Figure 4D**). MAP2K4-EE phosphorylated the kinase-dead p38 MAPK substrate albeit at a higher concentration compared to that of MAP3K1 activated and phosphorylated murine MAP2K4 (MAP2K4-AC).

### High throughput FTS screen discovers additional small molecules that inhibit MAP2K4

**Figure 5:**
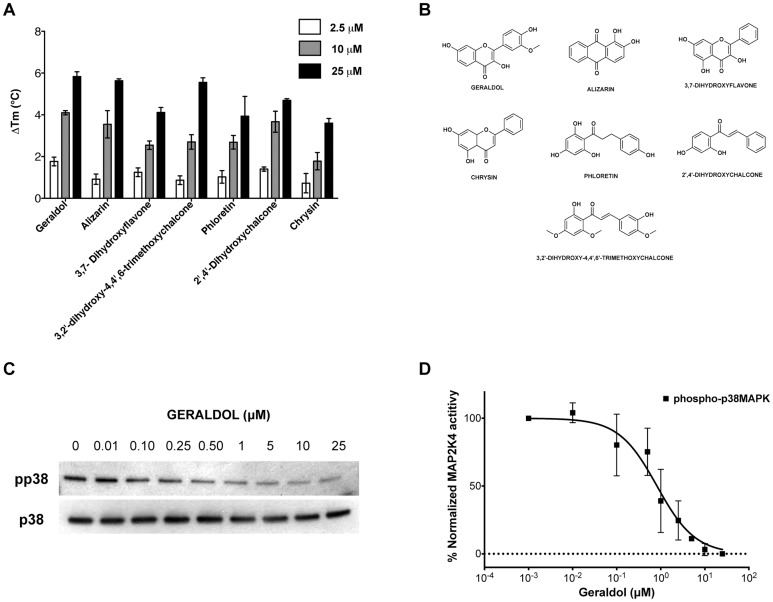
High-throughput screening yields seven additional MAP2K4 inhibitors. **A**. Graph of ΔTm values of Spectrum Collection hits determined at compound concentrations of 2.5, 10 and 25 µM. Values shown are the mean ± S.D (n = 3). **B**. Chemical structures of hit compounds. **C**. Geraldol inhibits MAP2K4 kinase. Representative Western blot using p38 MAPK as substrate is depicted **D**. densitometry measurements shown as mean ± S.D from two separate experiments are depicted along with the curve fit used for determining the IC_50_ value.

Having validated the technique on a focused kinase inhibitor library, a larger and more chemically diverse library was next selected for FTS screening. The Spectrum Collection of 2000 compounds from Microsource Discovery Systems consists of drug-like molecules (60%), natural products (25%) and other bioactive molecules (15%) and has been used by others in inhibitor discovery [35], [36]. It was initially screened using FTS in a high-throughput fashion followed by screening individual hits and ultimately yielded 7 compounds with ΔTm of at least 1.5°C at 10 µM. The ΔTm values for each of the 7 Spectrum Collection hit compounds at 2.5, 10 and 25 µM are depicted in **Figure 5A** representative raw melting curves are shown in **Figure S7**. Their structures are depicted in **Figure 5B** and a tabular comparison of their ΔTm at 10 µM and IC_50_ values using the p38 based kinase assay system are depicted in **Table 2**. Six of 7 hits were flavonoids, a broad class of plant secondary metabolites. These include geraldol (3,4′,7-Trihydroxy-3′-methoxyflavone), 3,7-dihydroxyflavone and chrysin (5,7-dihydroxyflavone) that thermally stabilized MAP2K4 up to 4.1°C, 2.5°C and 1.8°C at 10 µM, respectively. Three of the Spectrum Collection hits, phloretin or dihydrochalcone (ΔTm = 2.7°C), 3,2′-dihydroxy-4,4′,6-trimethoxychalcone (ΔTm = 2.7°C) and 2′,4′-dihydroxychalcone (ΔTm = 3.7°C), are members of the chalcone sub-class, within the flavonoid family. Alizarin (1,2-dihydroxyanthraquinone), a commonly used red-dye, also thermally stabilized the protein (ΔTm = 3.5°C). The ability of compounds identified by FTS to inhibit MAP2K4 kinase activity *in vitro* was then assessed. A representative kinase assay result showing the effect of Geraldol on p38 MAPK phosphorylation is shown in **Figure 5C** and the corresponding densitometry analysis is shown in **Figure 5D**. As shown in **Table 2** **and Figure S8**, all 7 hits inhibited MAP2K4 kinase activity with IC_50_ values ranging from 0.8-21.5 µM. It is to be noted that all 15 FTS identified hits from both libraries inhibited MAP2K4 activity in the in-vitro kinase assay and importantly Daidzein, a FTS identified non-binder did not.

**Table 2:** FTS and kinase assay data of hits from the Spectrum Collection.

Compound	ΔTm at 10 µM (°C)	IC_50_-p38 ( µM)
Geraldol	4.1±0.1	0.8 (0.5 to 1.2)
2′,4′-dihydroxychalcone	3.67±0.50	16.9 (6.8 to 42.1)
Alizarin	3.55±0.65	0.9 (0.6 to 1.4)
2′,3-dihydroxy-4,4′,6-trimethoxychalcone	2.70±0.35	21.5 (11.2 to 41)
Phloretin	2.69±0.33	12.3 (9.6 to 15.8)
3,7-dihydroxyflavone	2.55±0.20	0.8 (0.4 to 1.4)
Chrysin	1.78±0.42	10.1 (6 to 17.1)

Data shown as Mean ± SD with n = 3 for ΔTm. IC_50_ values are Mean (95% confidence intervals) calculated by fitting data averaged from replicates performed twice at separate times.

### Construction of a MAP2K4-ligand binding model

**Figure 6:**
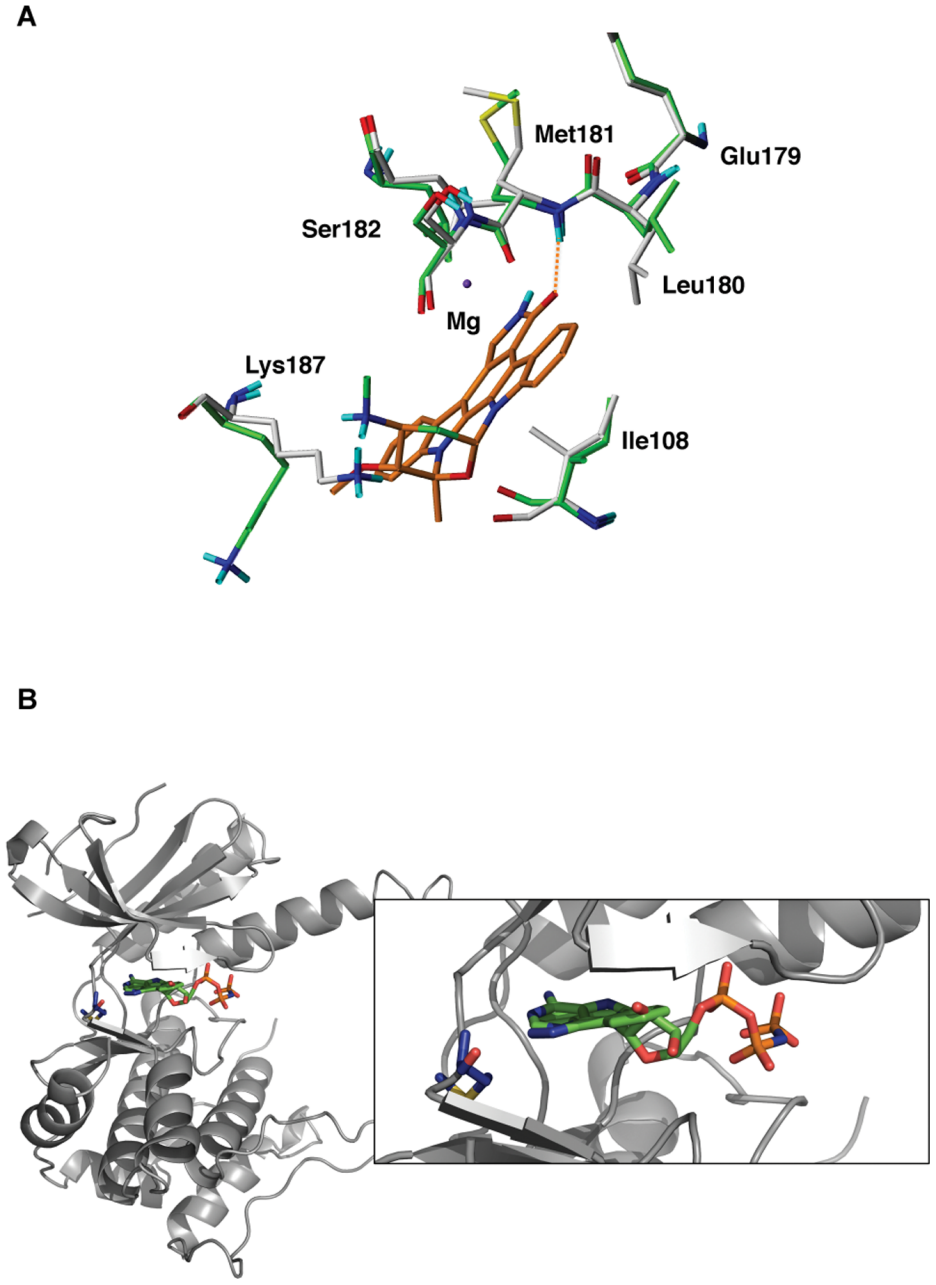
*In silico* docking predicts that all the hits bind MAP2K4's ATP site. **A**. An illustration of the Mutation based Induced Fit Docking is shown here with Staurosporine (Orange) and MAP2K4 residues that were mutated temporarily to alanine. Residues in final docked pose, i.e. post-mutation are colored green and those prior to the mutation step are colored in gray; the coordinating magnesium is shown in purple. **B**. The docked pose of SP600125 (green) with MAP2K4 is shown. The backbone NH of Met181 (blue) is predicted to form a hydrogen bond interaction is similar to one seen with AMP-PNP (orange).

Docking studies were pursued in order to gain insight into how inhibitors may interact with MAP2K4. Eleven hits (8 from the ScreenWell Kinase Inhibitor library and 3 representative hits from the Spectrum Collection) were docked to the 3D structure of MAP2K4 (PDB: 3ALO), using the Induced Fit Docking (IFD) method as described in Methods. Preliminary analysis revealed that 3 hits, Staurosporine, PP1 and PP2, did not dock to the MAP2K4 ATP binding site due to the non-complementarity of shape and electrostatic parameters between the active site residues and the ligands. In the case of Staurosporine, Lys187, Leu 180 and Ile 108 were restricting the entry of the ligand (**Figure 6A**). Similarly for PP1 and PP2, the residues Lys131, Asp247 and Leu 236 were preventing the entry of the compounds into the active site of MAP2K4. These residues were mutated to alanine temporarily and the ligand prepositioned into the active site with a softer Van der Waal potential and its energy minimized using the OPLS-2005 force field. The residues were then mutated back and applying the most stringent Van der Waals and electrostatic constraints the complex was once again minimized in the OPLS-2005 force field. During this process, the side chains of the active site residues were perturbed without changing the backbone (C-α). In this manner, the mutation-based IFD implementation was used to generate MAP2K4 docking poses for Staurosporine, PP1 and PP2.

The poses yielding the highest Glide-XP scores for the eleven MAP2K4 inhibitors were selected for, and the apparent binding and ligand strain energies in this conformation were computed (**Table 3**), as described in the Methods. All the inhibitors were predicted to bind to the MAP2K4 ATP pocket and formed a hydrogen-bond interaction with the backbone carbonyl and/or amide of the methionine 181 in the hinge region of the kinase, a previously established kinase interaction with inhibitors [37]. In **Figure 6B**, the docked pose of SP600125 bound to MAP2K4 is superimposed with the AMP-PNP molecule from the structure used in the docking experiment. The N1 nitrogen of SP600125 is 2.9 Å away from the backbone amide group of Met 181, indicative of a hydrogen bond interaction between the two. A similar H-bonding interaction is seen with the N1 of the adenine ring of AMP-PNP with the Met 181 backbone carbonyl. Considering the docked poses of two structurally similar compounds, Apigenin and Quercetin, highlights the refining capability of the model. It was observed that the two additional –OH groups of Quercetin sterically clash into the protein active site residues and the meta position –OH of the terminal phenyl group of Quercetin unfavorably interacts with the backbone carbonyl of G109. In the absence of crystal structures of MAP2K4 with the identified inhibitors, the in silico model provides a framework to visually characterize the underlying basis of our experimental findings.

**Table 3:** Binding parameters determined from in silico analysis of MAP2K4 inhibitors.

Compound	Binding Energy (kcal/mol)	Ligand Strain Energy (kcal/mol)
Staurosporine	−15.41	11.25
SP600125	−16.97	3.60
PP1	−13.57	6.25
PP2	−14.01	2.36
Apigenin	−15.67	7.14
AG825	−11.07	5.29
Phloretin	−13.22	1.26
5-iodotubercidin	−12.05	6.52
Alizarin	−11.95	3.25
Geraldol	−8.12	2.50
Quercetin	−7.09	16.20

Binding Energy values shown are for final inhibitor docked pose/solution. The Ligand Strain Energy is the difference in energy between the ligand in an energy-minimized state outside of the receptor and the optimized energy of the ligand in the binding pocket.

## Conclusion

The metastatic cascade has long been recognized as a therapeutic target of high potential and clinical impact. Through our collaborative research platform investigating metastasis, we have identified the importance of MAP2K4 kinase, both biologically and therapeutically as a target. To the best of our knowledge, highly selective and potent inhibitors of MAP2K4 have not been reported. In the current study, we have created an efficient and robust platform for the identification of novel inhibitors of MAP2K4 kinase activity. Our provision of this platform provides an informed pathway for the rational design and discovery of optimized MAP2K4 inhibitors.

An adaptation of the FTS assay approach has provided a quick and low cost primary screen to both select out kinase inhibitors that did not interact with MAP2K4, and importantly, to identify those that bound and thermally stabilized the protein. The assay's amenability to high throughput screening meant that we were able to successfully scale up our in-house FTS assay and implement it on a much larger chemical library. By coupling this to a second tier *in* vitro MAP2K4 kinase assay, employing physiologically relevant substrates, we then ascertained that all compounds identified by the FTS assay as MAP2K4 binders also inhibited its activity. Finally, our construction of a MAP2K4 model of bound ligand and its ability to closely predict FTS behavior provides a three dimensional framework for future experimental work. It thereby serves as an important starting point for creating novel MAP2K4 inhibitors, understanding their potential to bind the protein, and will aid their future refinement.

Our investigations, and by extension, the platform we have described has some important limitations. Though FTS has the potential to identify ligands that bind outside of the ATP pocket, we have not yet demonstrated this capability in part due to a deliberately high threshold set by us. On a theoretical basis, our platform is well poised to identify them. Such compounds would be identified as positive hits on FTS screening, and may or may not inhibit kinase activity *in vitro*. The former situation would be easy to ascertain, but the latter would not be readily apparent. However, it would likely not fit well into our three-dimensional model, and would require relatively extensive physical and biochemical based analysis. Also, it is important to consider that, as we have not yet determined the crystal structure of MAP2K4 with these inhibitors. Therefore our proposed model is based upon an *in silico* analysis, and needs to be interpreted with a high degree of caution. Like all structural models that have not yet been experimentally confirmed, ours should be considered a framework upon which refinements and enhancements can be added. Finally, human MAP2K4-EE protein was used in FTS assays, differs from the murine wild type phosphorylated MAP2K4 protein used in *in vitro* kinase assays, and has the potential to induce error. Given that there is 98% sequence homology between human and murine MAP2K4, and that our findings demonstrate concordance between the performance of human MAP2K4 in FTS assays and murine MAP2K4 in *in vitro* kinase assays, this potential appears to be low.

In summary, in the context of the biologically and therapeutically important MAP2K4, we have provided a platform that serves to understand the relationship between the kinase and ligands. Further, it provides an avenue for the future discovery and validation of MAP2K4 inhibitors.

## Supporting Information

Figure S1 **MAP2K4 constructs used in this study shown along with full-length protein (MAP2K4-WT).** FTS assays primarily employed human MAP2K4-EE (S257E, T261E) and the kinase domain construct (MAP2K4-KD) was utilized for the in-silico docking analysis. MAP3K1 activated murine MAP2K4 (MAP2K4-AC) was used in the in-vitro kinase assays.
(TIF)

Figure S2 **Representative raw melting curves of MAP2K4-EE with 1 mM of ATP/related compounds.** Select thermograms corresponding to one of three replicates from histogram data shown in Fig 2B are displayed here. The overall change in fluorescence intensity (arbitrary units) is shown in the y-axis along with temperature (°C) in the x-axis. The portion of the curve colored in green was utilized for the Boltzmann curve fit.
(TIF)

Figure S3 **Representative raw melting curves of MAP2K4-EE with hits from the ENZO kinase inhibitor library at 10 µM.** Select thermograms of hits from the Enzo kinase inhibitor library are displayed here. Also shown are representative conditions corresponding to no compound (HEPES), DMSO (control) and Daidzein (non-binder). The overall change in fluorescence intensity (arbitrary units) is shown in the y-axis along with temperature (°C) in the x-axis. The portion of the curve colored in green was utilized for the Boltzmann curve fit.
(TIF)

Figure S4 **Compounds that bind MAP2K4-EE also bind the MAP2K4-KD construct.** Graph of ΔTm values of ScreenWell Kinase Inhibitor library hits determined at 10 µM. Values shown are the mean ± S.D (n = 2).
(TIF)

Figure S5 ***In-vitro*** **kinase assays using inhibitors identified from the ScreenWell Kinase Inhibitor library and JNK1(K55M) as protein substrate.** Phospho-protein and total protein antibody blots are shown at indicated concentration of inhibitor. Experiments were repeated twice at separate times and one representative blot for each inhibitor is shown here.
(TIF)

Figure S6 ***In-vitro*** **kinase assays using inhibitors identified from the ScreenWell Kinase Inhibitor library and p38 MAPK(K53A) as protein substrate.** Phospho-protein and total protein antibody blots are shown at indicated concentration of inhibitor. Experiments were repeated twice at separate times and one representative blot for each inhibitor is shown here.
(TIF)

Figure S7 **Representative raw melting curves of MAP2K4-EE with hits from the Spectrum Collection at 10 µM.** Select thermograms of hits from the Spectrum Collection are displayed here. Also shown are representative conditions corresponding to no compound (HEPES) and equivalent DMSO (control). The overall change in fluorescence intensity (arbitrary units) is shown in the y-axis along with temperature (°C) in the x-axis. The portion of the curve colored in green was utilized to generate normalized thermograms.
(TIF)

Figure S8 ***In-vitro*** **kinase assays using inhibitors identified from the Spectrum Collection.** p38 MAPK(K53A) was used as protein substrate and phospho-protein and total protein antibody blots are shown at indicated concentration of inhibitor. Experiments were repeated twice at separate times and one representative blot for each inhibitor is shown here.
(TIF)

Table S1 Δ**Tm of Enzo kinase inhibitor library compounds at 10 µM vs MAP2K4-EE.**
(TIF)
