# Correction: A Fluorescence-Based Thermal Shift Assay Identifies Inhibitors of Mitogen Activated Protein Kinase Kinase 4

**DOI:** 10.1371/annotation/5edeb1de-b76c-4fd9-a3f6-0f1cf45e3905

**Published:** 2013-12-23

**Authors:** Sankar N. Krishna, Chi-Hao Luan, Rama K. Mishra, Li Xu, Karl A. Scheidt, Wayne F. Anderson, Raymond C. Bergan

This article has been republished to correct the funding statement. 

