# Supplementary figures and images for: A Fluorescence-Based Thermal Shift Assay Identifies Inhibitors of Mitogen Activated Protein Kinase Kinase 4
Source: PLoS One. 2013 Dec 5;8(12):e81504. doi: 10.1371/journal.pone.0081504 (PMC3855329; doi:10.1371/journal.pone.0081504)

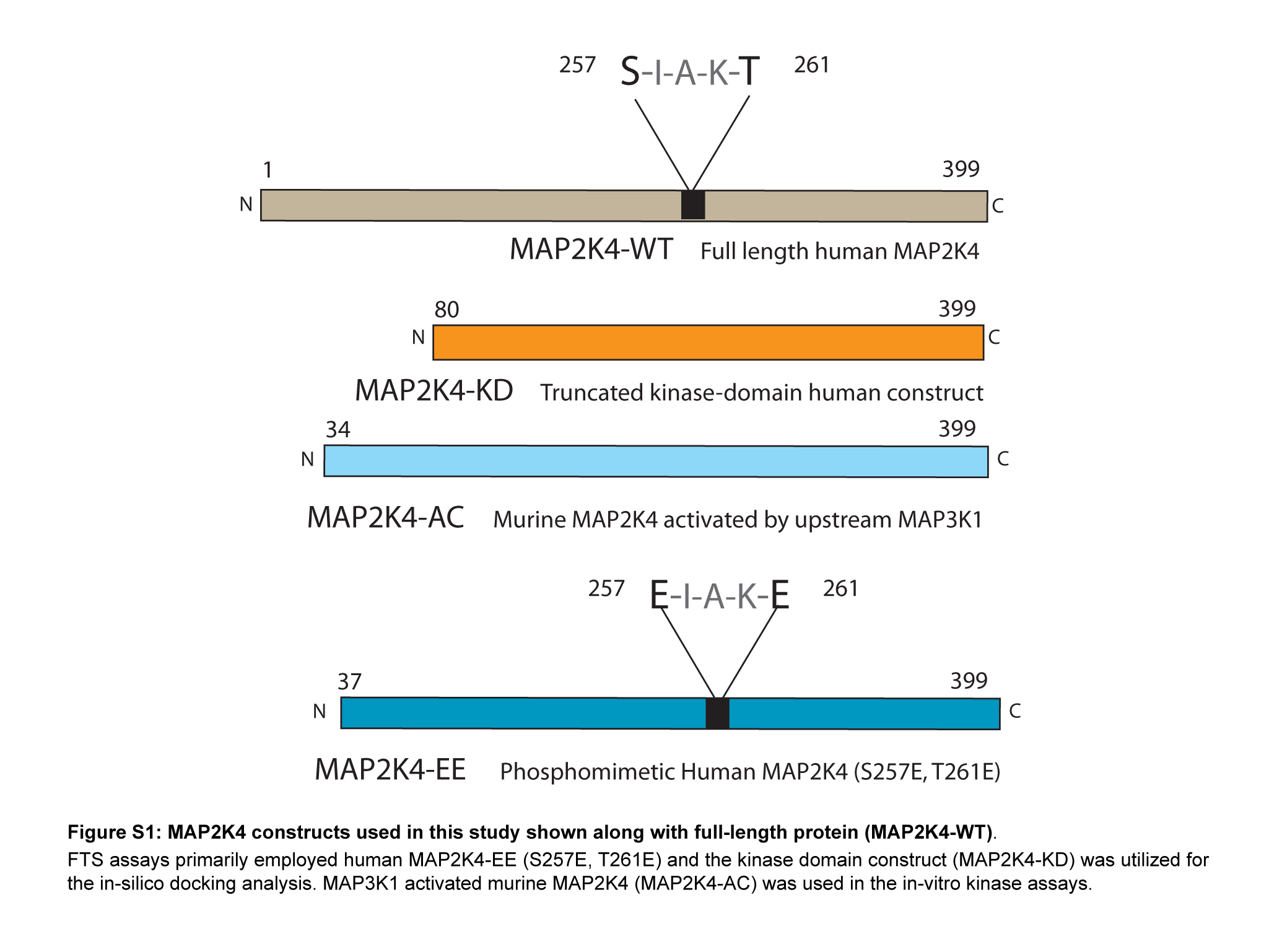

Supplement: Figure S1 — MAP2K4 constructs used in this study shown along with full-length protein (MAP2K4-WT). FTS assays primarily employed human MAP2K4-EE (S257E, T261E) and the kinase domain construct (MAP2K4-KD) was utilized for the in-silico docking analysis. MAP3K1 activated murine MAP2K4 (MAP2K4-AC) was used in the in-vitro kinase assays. (TIF) [file pone.0081504.s001.tif]

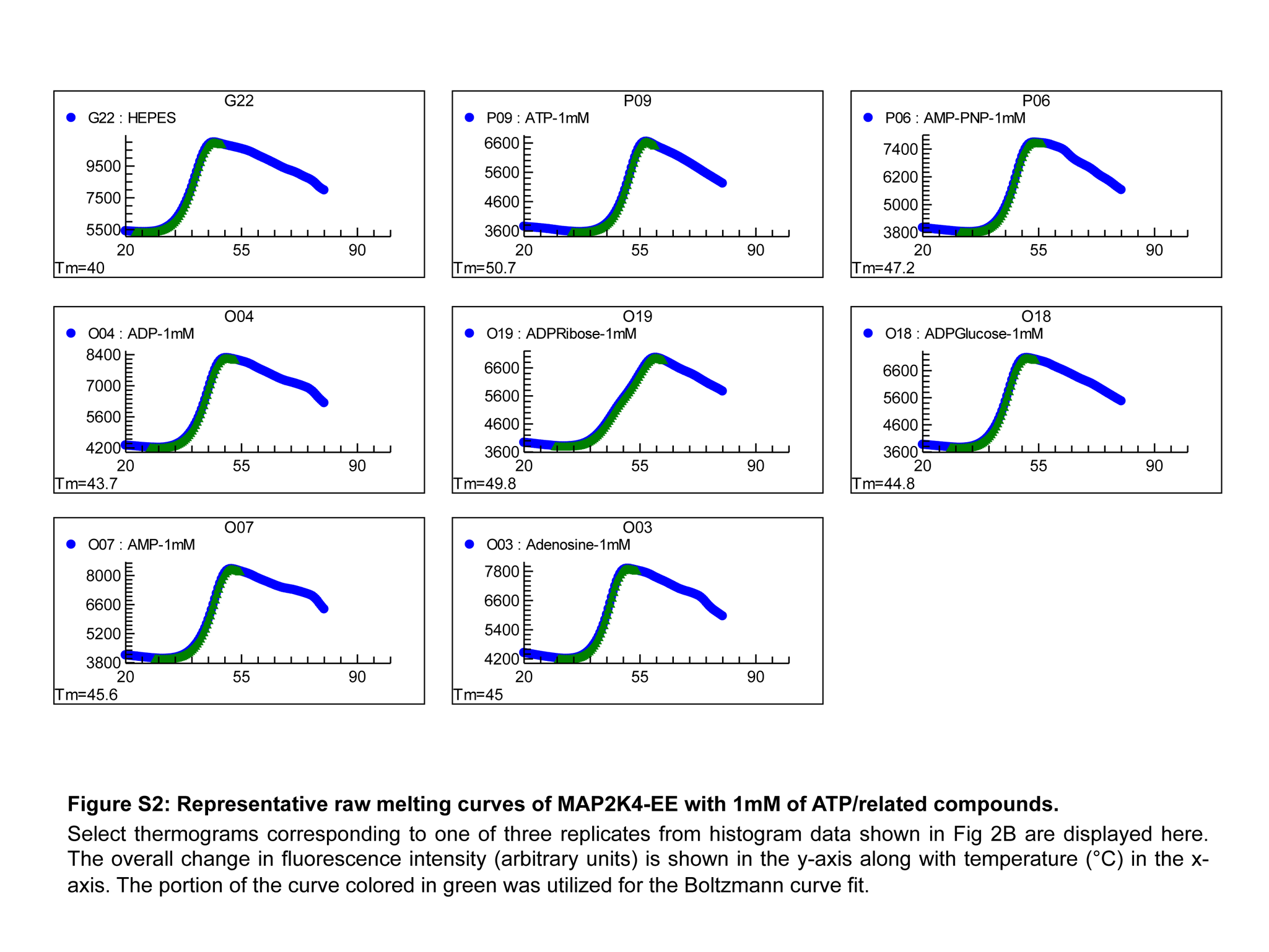

Supplement: Figure S2 — Representative raw melting curves of MAP2K4-EE with 1 mM of ATP/related compounds. Select thermograms corresponding to one of three replicates from histogram data shown in Fig 2B are displayed here. The overall change in fluorescence intensity (arbitrary units) is shown in the y-axis along with temperature (°C) in the x-axis. The portion of the curve colored in green was utilized for the Boltzmann curve fit. (TIF) [file pone.0081504.s002.tif]

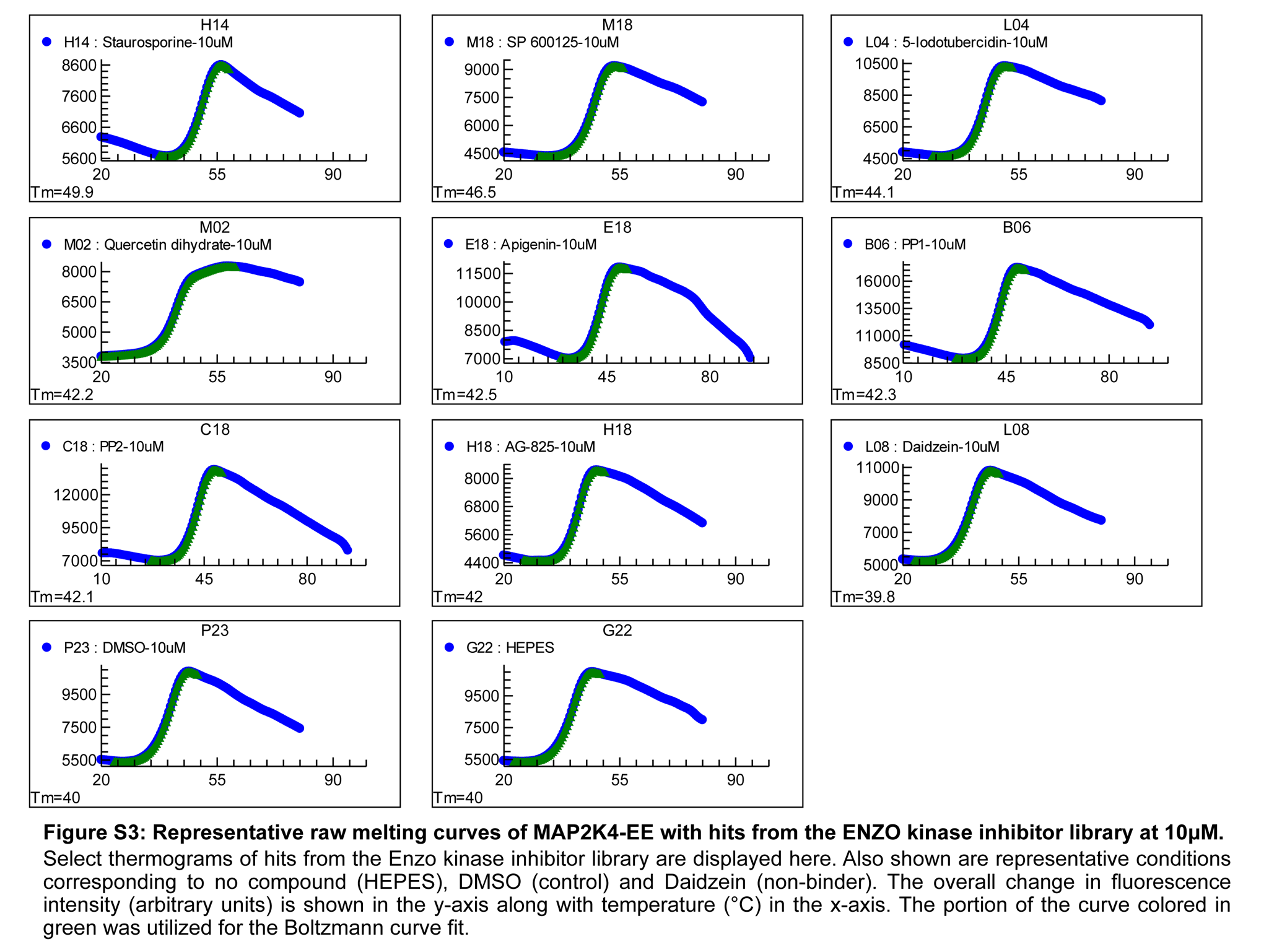

Supplement: Figure S3 — Representative raw melting curves of MAP2K4-EE with hits from the ENZO kinase inhibitor library at 10 µM. Select thermograms of hits from the Enzo kinase inhibitor library are displayed here. Also shown are representative conditions corresponding to no compound (HEPES), DMSO (control) and Daidzein (non-binder). The overall change in fluorescence intensity (arbitrary units) is shown in the y-axis along with temperature (°C) in the x-axis. The portion of the curve colored in green was utilized for the Boltzmann curve fit. (TIF) [file pone.0081504.s003.tif]

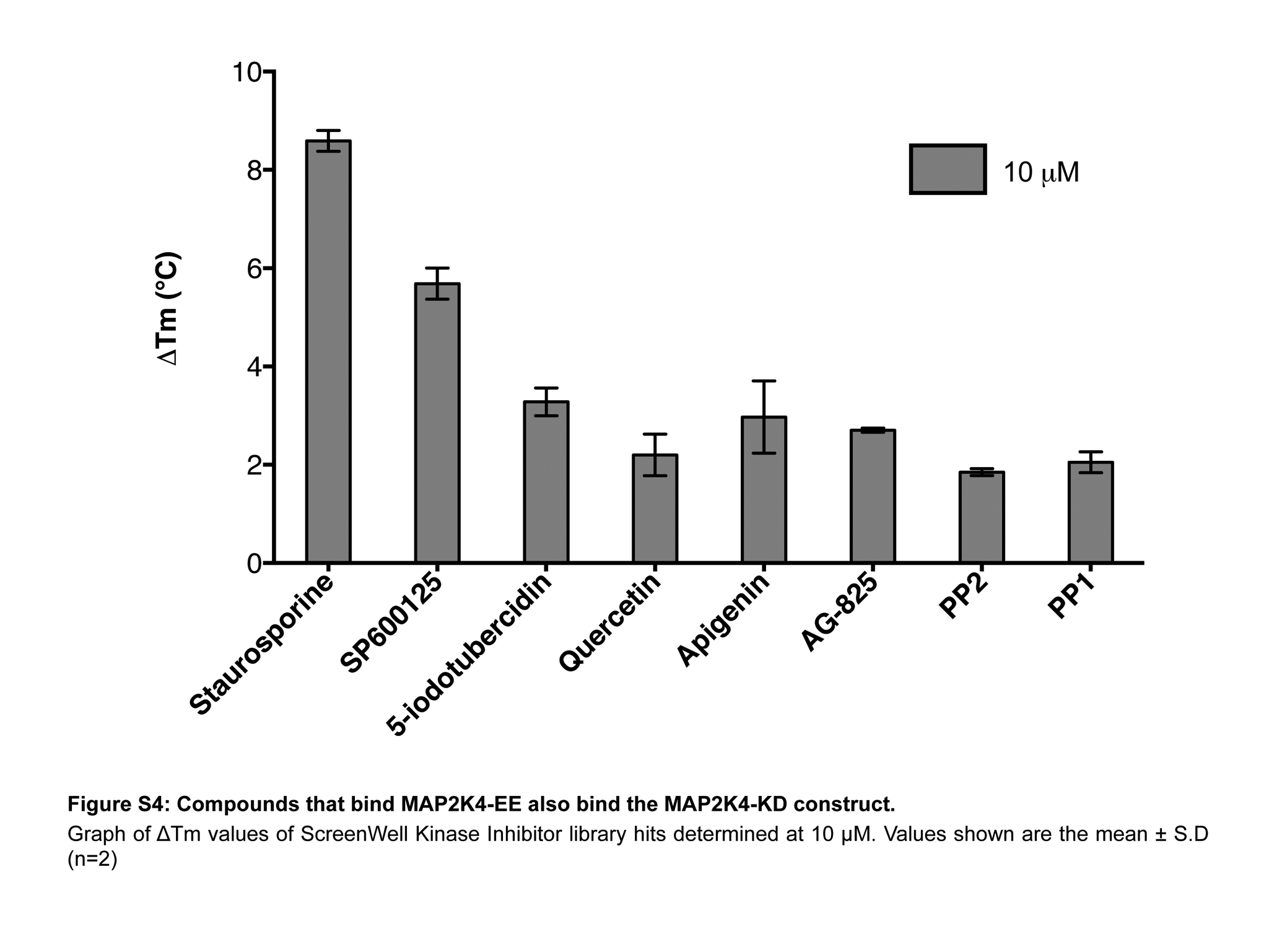

Supplement: Figure S4 — Compounds that bind MAP2K4-EE also bind the MAP2K4-KD construct. Graph of ΔTm values of ScreenWell Kinase Inhibitor library hits determined at 10 µM. Values shown are the mean ± S.D (n = 2). (TIF) [file pone.0081504.s004.tif]

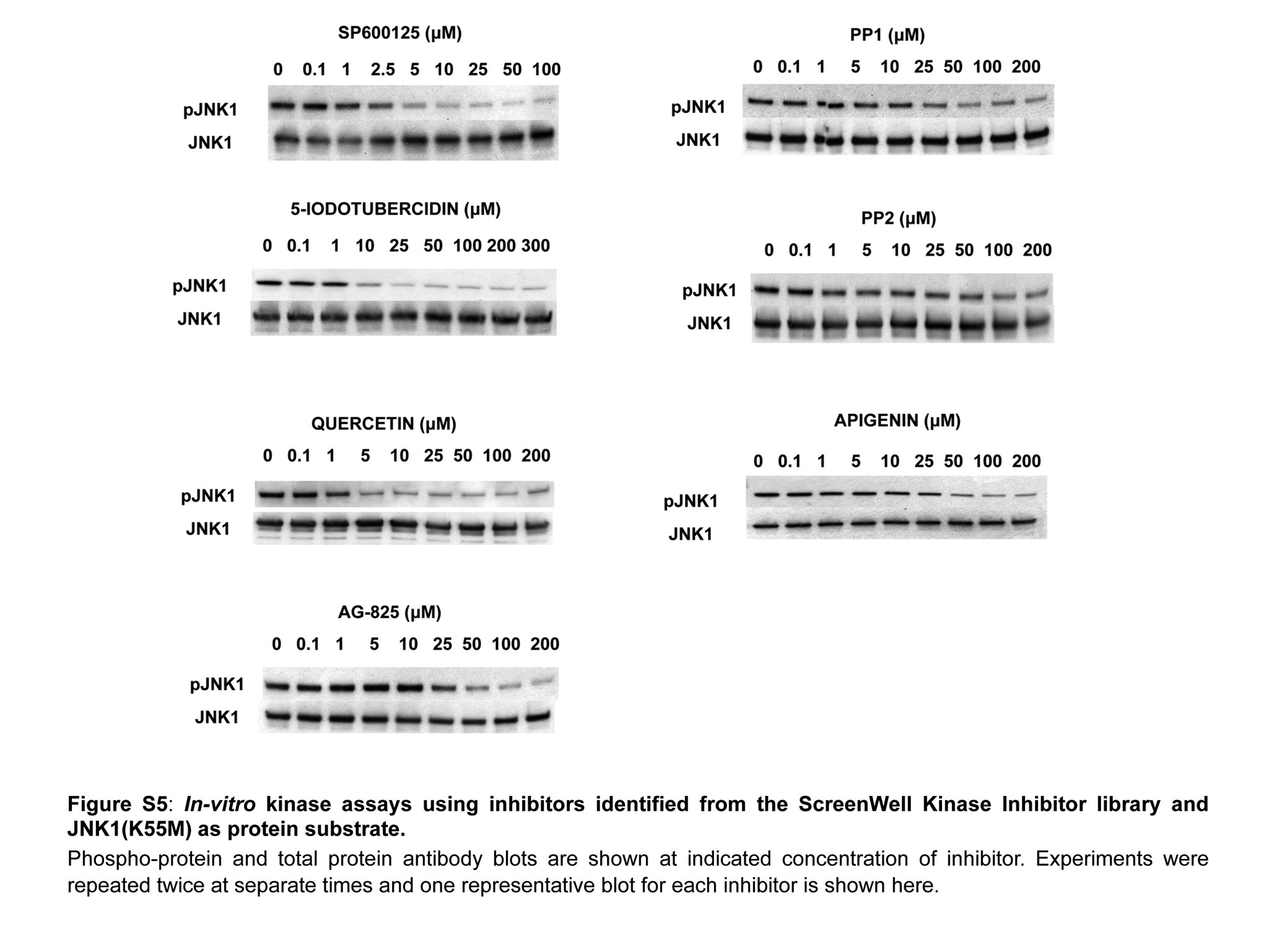

Supplement: Figure S5 — In-vitro kinase assays using inhibitors identified from the ScreenWell Kinase Inhibitor library and JNK1(K55M) as protein substrate. Phospho-protein and total protein antibody blots are shown at indicated concentration of inhibitor. Experiments were repeated twice at separate times and one representative blot for each inhibitor is shown here. (TIF) [file pone.0081504.s005.tif]

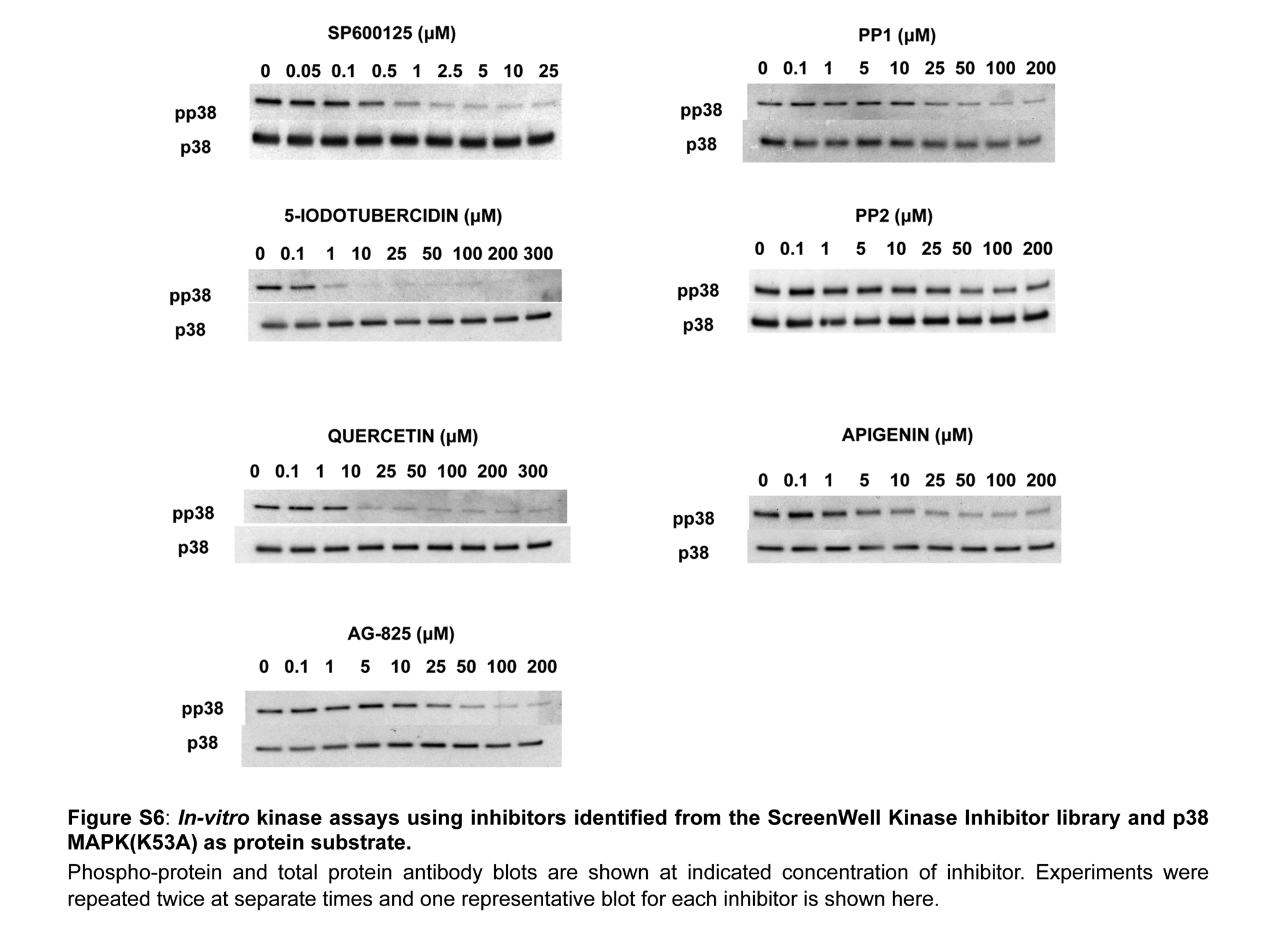

Supplement: Figure S6 — In-vitro kinase assays using inhibitors identified from the ScreenWell Kinase Inhibitor library and p38 MAPK(K53A) as protein substrate. Phospho-protein and total protein antibody blots are shown at indicated concentration of inhibitor. Experiments were repeated twice at separate times and one representative blot for each inhibitor is shown here. (TIF) [file pone.0081504.s006.tif]

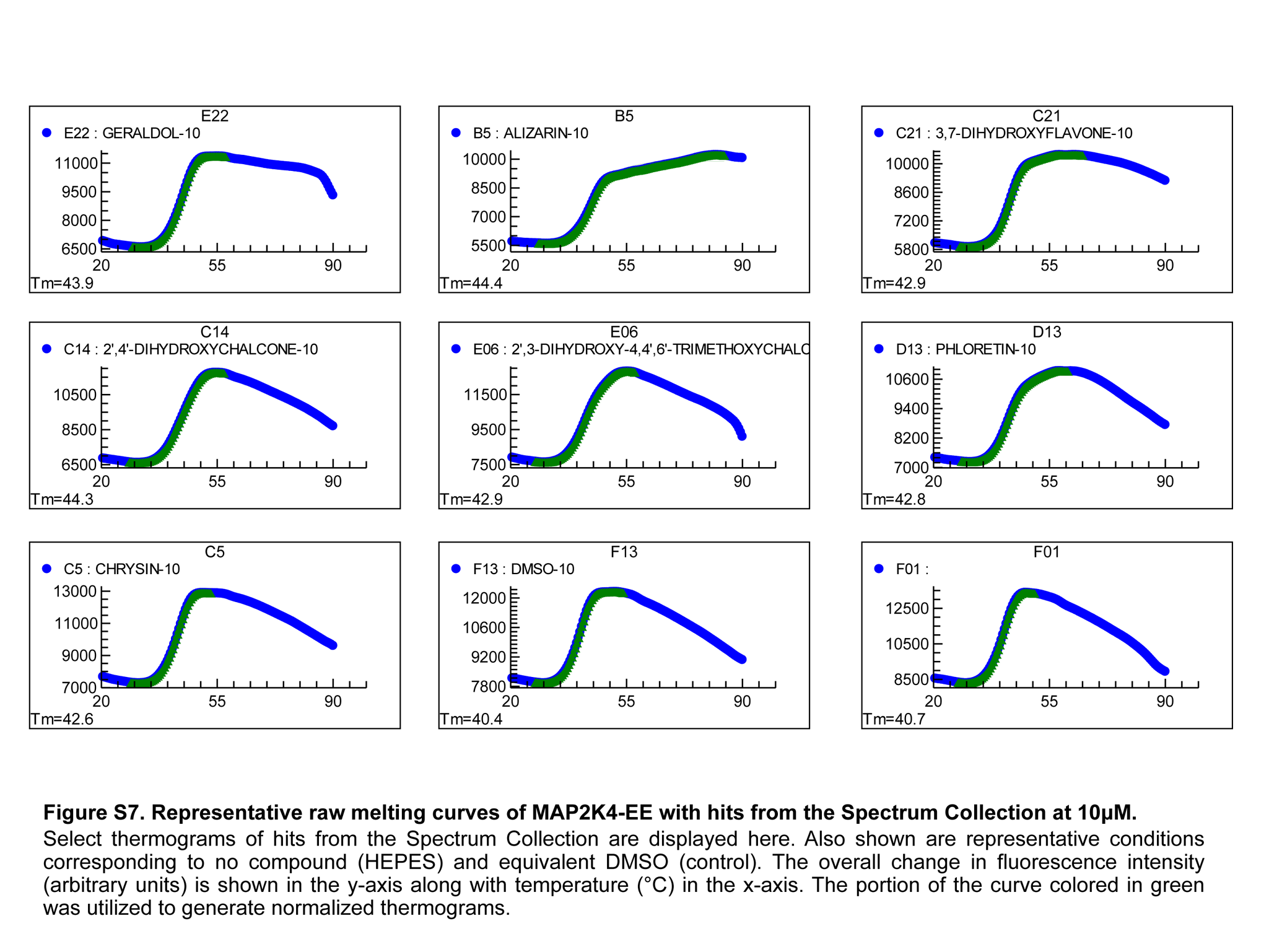

Supplement: Figure S7 — Representative raw melting curves of MAP2K4-EE with hits from the Spectrum Collection at 10 µM. Select thermograms of hits from the Spectrum Collection are displayed here. Also shown are representative conditions corresponding to no compound (HEPES) and equivalent DMSO (control). The overall change in fluorescence intensity (arbitrary units) is shown in the y-axis along with temperature (°C) in the x-axis. The portion of the curve colored in green was utilized to generate normalized thermograms. (TIF) [file pone.0081504.s007.tif]

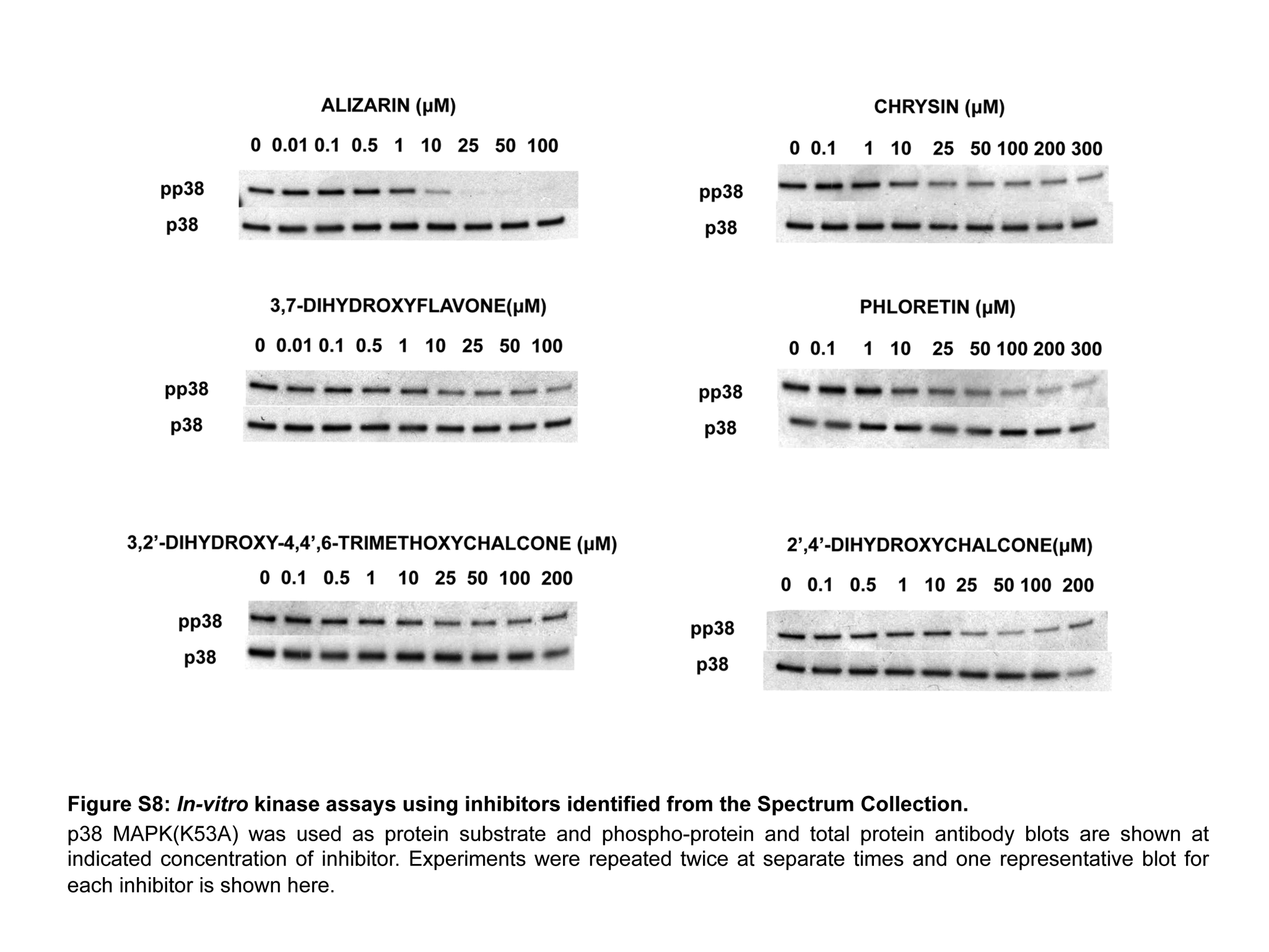

Supplement: Figure S8 — In-vitro kinase assays using inhibitors identified from the Spectrum Collection. p38 MAPK(K53A) was used as protein substrate and phospho-protein and total protein antibody blots are shown at indicated concentration of inhibitor. Experiments were repeated twice at separate times and one representative blot for each inhibitor is shown here. (TIF) [file pone.0081504.s008.tif]

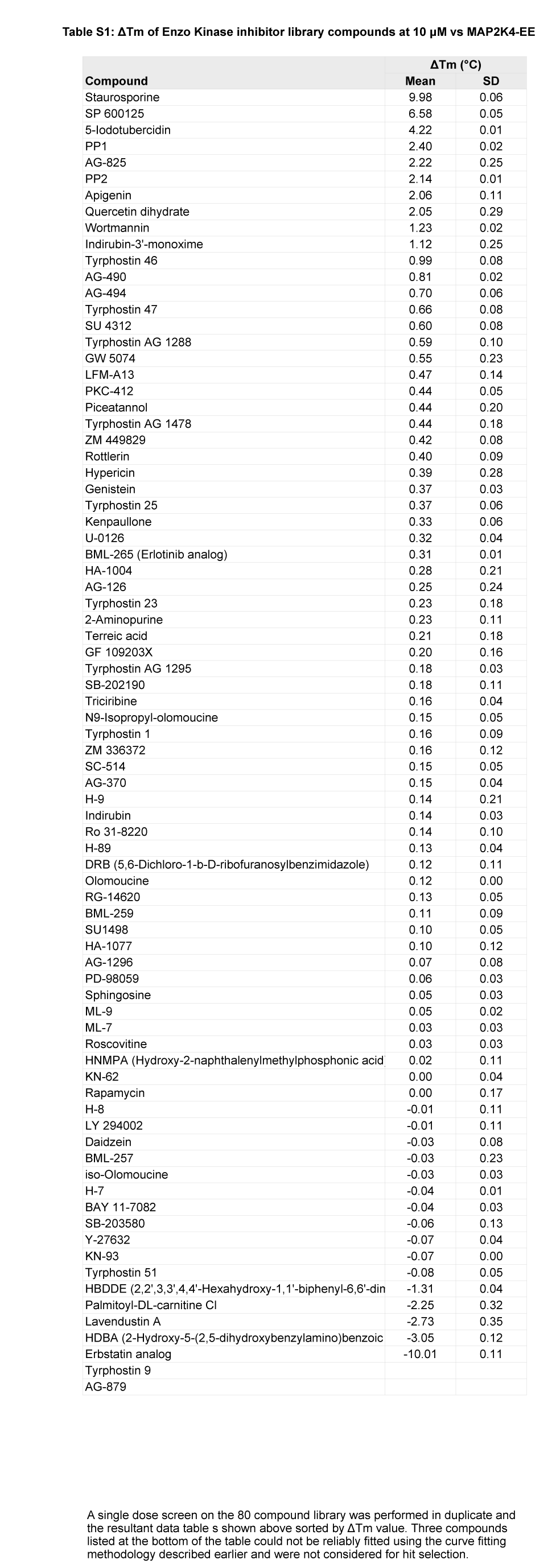

Supplement: Table S1 — ΔTm of Enzo kinase inhibitor library compounds at 10 µM vs MAP2K4-EE. (TIF) [file pone.0081504.s009.tif]
